# A technique for analyzing the variability of activation thermodynamic and solvent model parameters†

**DOI:** 10.1039/d4ra07211a

**Published:** 2025-02-06

**Authors:** Floyd L. Wiseman, Dane W. Scott

**Affiliations:** a Blue Mountain Christian University, Department of Mathematics & Natural Science P.O. Box 160 Blue Mountain MS 38610 USA; b East Tennessee State University, Department of Chemistry 325 Treasure Lane Johnson City TN 37614 USA

## Abstract

The fundamental thermodynamic equation of chemical kinetics has recently been used to analyze rate data from the hydrolysis reaction of *tert*-butyl chloride in the acetonitrile/water solvent system. Although this study showcased the deeper level of insight afforded from the fundamental equation, at the time of the publication no technique had been developed for analyzing the functional dependencies of the activation thermodynamic and solvent model parameters. We have since developed a three-step technique briefly described as follows. The first step includes conducting a linear regression analysis using a linearized form of the fundamental equation to determine if the parameters are constant. The second step includes a technique for evaluating the functional forms of the parameters if they are not constant, and the third step includes a technique for constructing parameter grid equations. The three-step analysis has been applied to some of the rate data from our studies on the *tert*-butyl chloride hydrolysis reaction. The results show the intrinsic activation entropy and Kirkwood–Onsager parameter depend on the electrostatic environment of the bulk solvent and the close-range interactions associated with the solvation shell. Auxiliary topics also presented in this article include an analysis showing mathematical expressions for intrinsic parameters cannot be evaluated, a discussion on the modeling benefits of the fundamental equation, and presentation of an empirical expression that correlates the solvent mole fraction term with the effect of the solvation shell.

## Abstract 2

The fundamental thermodynamic equation of chemical kinetics has recently been used to analyze rate data from the hydrolysis reaction of *tert*-butyl chloride in the acetonitrile/water solvent system.

## Graphical abstract

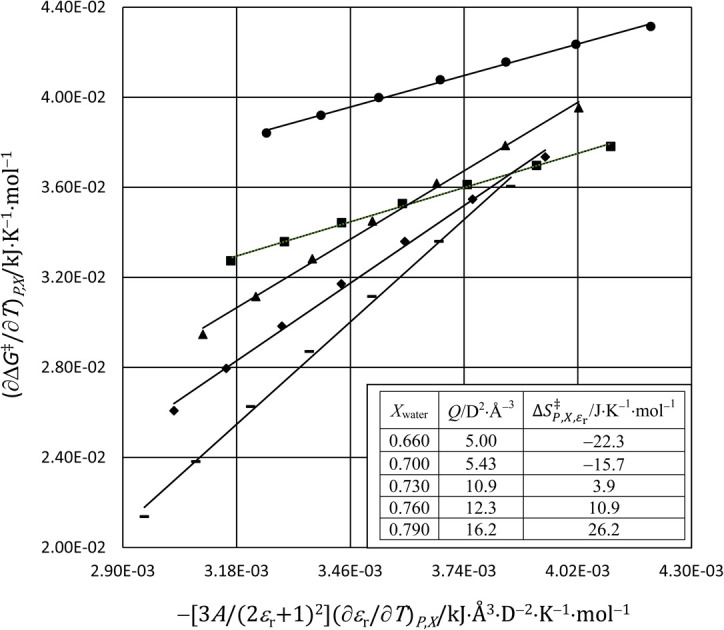

## Introduction

In some of our previous publications we have introduced and discussed the fundamental thermodynamic equation of chemical kinetics for binary solvents (the “fundamental equation” for short),^1–3^ and recently used it to analyze the hydrolysis reaction of *tert*-butyl chloride in the acetonitrile/water solvent system.^4^ This reaction was chosen because it has been well studied and the accepted mechanism is simple, which is a desired feature for interpreting rate data. The acetonitrile/water solvent system was chosen as this system has been well studied. We studied the reaction under isobaric/iso-mole fraction, isobaric/isodielectric, and isobaric/isothermal conditions, and the analyses yielded average values for the intrinsic activation entropy and solvent model parameters (we define these terms in the Theory section). While this study provided a successful proof of concept, at the time of the publication we had not developed a technique for systematically analyzing functional dependencies for the parameters. Since then, we have formulated and tested a three-step technique that assesses whether the parameters vary, and analyzes their functional forms if they do. In this article we present this technique in detail, and apply it to some of the rate data from the *tert*-butyl chloride hydrolysis studies. As we will show, this three-step technique uncovers some fascinating insight into the activation dynamics and how the two distinct regions of the solvent (the solvation shell and the bulk solvent) uniquely affect the reaction.

We briefly describe the three-step technique as follows. The first step includes conducting a linear regression analysis using a linearized form of the fundamental equation. If the plot is linear, the parameters are constant and the analysis is complete. If not, the process continues with the second step, which includes a “two-point” analysis that evaluates the parameters between all pairs of adjacent points in the data set. This creates a set of values for the parameters that can be cast as functions in any one of the domain variables. The final step, which is not always feasible for reasons we will discuss, includes conducting layered polynomial regression analyses to generate the parameter grid equations. This is the same technique used to generate the binary solvent grid equations (basically an equation of state). As the discussion unfolds, we use certain terms and phrases that we define as they are introduced.

We also present three auxiliary topics that are not fully discussed in any of our previous publications. These include an analysis that shows intrinsic parameters cannot be mathematically evaluated, a discussion of the modeling benefits of the fundamental equation, and the presentation of an empirical expression that correlates the mole fraction term with the effect of the solvation shell. These topics are not germane to the primary intent of this article, and are presented in ESI† Sections 1–3, respectively.

## Theory

### Introduction and definitions

The fundamental thermodynamic equation of chemical kinetics for a binary solvent is:^1–3^1
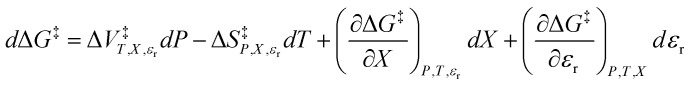
in which *P*, *T*, *X* (the mole fraction of either solvent component), and *ε*_r_ (the relative permittivity) comprise the complete set of system (or state) variables. In referencing eqn (1) and other activation thermodynamic equations, we will use the following definitions. The differential terms on the right-hand-side of the equation are the equation terms; Δ*G*^‡^, 
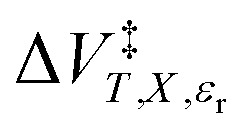
, and 
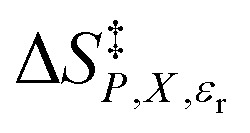
 are activation thermodynamic parameters; 
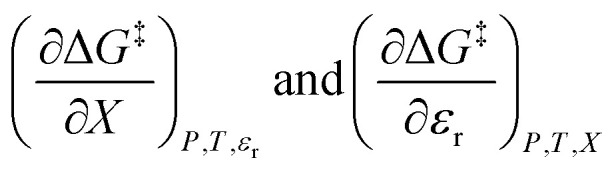
 are the mole fraction and electrostatic terms, respectively, and collectively they are the solvent model terms; and 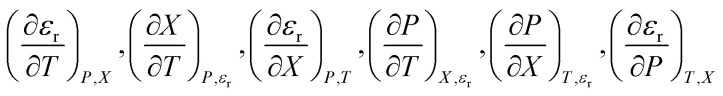, and their reciprocals are solvent terms. The solvent terms are all interrelated through the solvent grid equation, and are independent of the reaction system provided the reactants are in very small amounts.

When analyzing solvent effects, the solvent model terms are replaced with either theoretical or empirical equations. Theoretical equations contain parameters that have structural significance, but the parameters associated with empirical equations do not correlate with any structural feature. As they yield more interpretive results, theoretical equations are preferred and used if available.

Inasmuch as eqn (1) is a true thermodynamic equation, each activation thermodynamic parameter or solvent model term correlates an aspect of the activation process to a system variable. The first two right-hand-side terms correlate the intrinsic activation volume, 
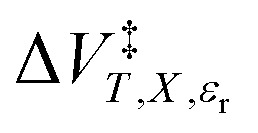
, with the pressure, and the intrinsic activation entropy, 
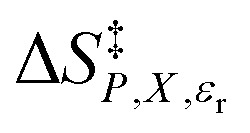
, with the temperature. An intrinsic parameter is one in which all complementary variables are constant. A complementary variable is any system variable other than the one correlated with the activation parameter or solvent model term (*e.g. T*, *X*, and *ε*_r_ for 
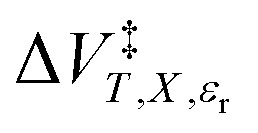
), and the primary variable is the one that is correlated (*e.g. P* for 
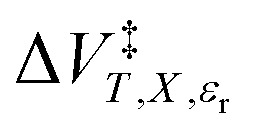
). The electrostatic term, 
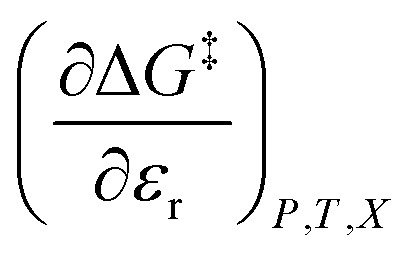
, correlates with the relative permittivity, and as we will show later, the mole fraction term, 
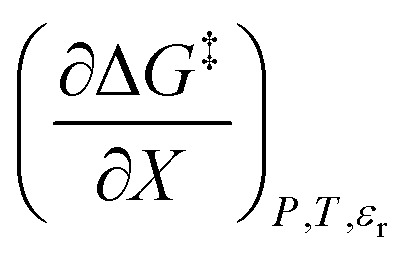
, correlates the mole fraction with the short-range solvent–solute interactions associated with the solvation shell.

Eqn (1) has one more term than prescribed by the phase rule. However, analyzing an electrostatic equation, or any other intrinsic term in eqn (1), requires there be as many equation terms as system variables. This critical point can be illustrated by dropping the electrostatic term, which renders the following equation:2
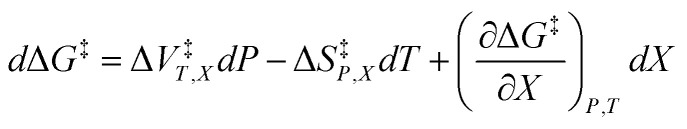


As eqn (2) has no *explicit* relative permittivity term, it cannot be used to analyze an electrostatic equation. However, eqn (2) is related implicitly to the relative permittivity, as illustrated in the following set of partial differential expressions:3
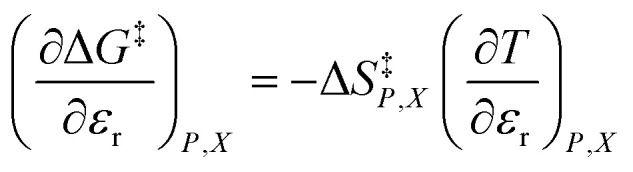
4
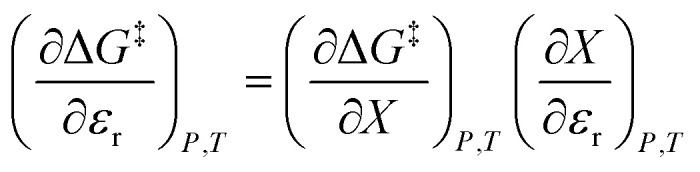
5
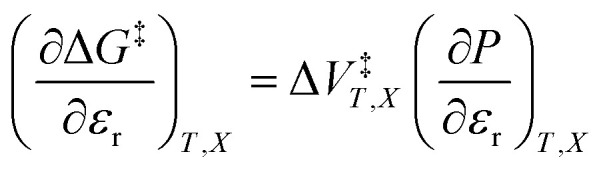


The implicit dependency vanishes if the solvent term is zero.

We can compare eqn (3)–(5) with the following corresponding expressions from eqn (1):6
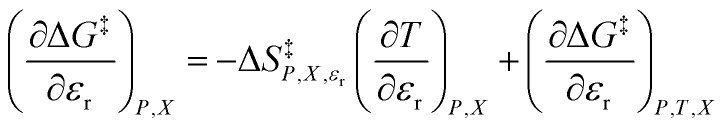
7
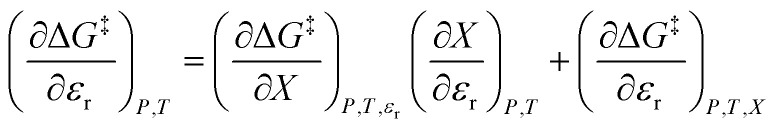
8
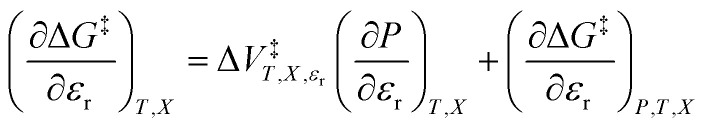


The electrostatic term, 
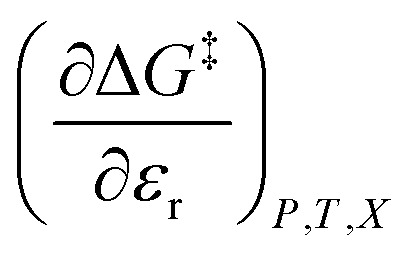
, is the only explicit term in relative permittivity, and is the only term that can be used to model an electrostatic equation. As these equations all have two right-hand-side terms, the electrostatic term, like any of the terms in eqn (1), is always analyzed in conjunction with another term.

### The three-step analysis for generating the parameter functionalities and the parameter grid equations

In the following subsections we outline the three-step analysis in detail. But first we briefly discuss the experimental protocol required for conducting the analysis.

### Experimental protocol for generating the data required for a three-step analysis

Conducting a three-step analysis requires rate data for which all variables but one in the variable space are systematically varied. The variable that is not systematically varied is functionally related to the other variables *via* the appropriate solvent grid equation. Our kinetic analyses always include a set of isobaric experiments for which the temperature and mole fraction are systematically varied. The experimental grid set for these conditions can be represented using set notation as {*T*_*i*_[*X*_*j*_(*ε*_r,*i*,*j*_)]} or {*X*_*i*_[*T*_*j*_(*ε*_r,*i*,*j*_)]}, in which *ε*_r,*i*,*j*_ is related to *X_j_* and *T_i_* by the solvent grid equation. As an example, suppose we conduct isobaric rate measurements at four temperatures, and for each temperature we conduct iso-mole fraction rate measurements at five mole fractions. The elements in the grid set are {*T*_1_[*X*_1_(*ε*_r,1,1_), *X*_2_(*ε*_r,1,2_), *X*_3_(*ε*_r,1,3_), *X*_4_(*ε*_r,1,4_), *X*_5_(*ε*_r,1,5_)]; *T*_2_[*X*_1_(*ε*_r,2,1_), *X*_2_(*ε*_r,2,2_), *X*_3_(*ε*_r,2,3_), *X*_4_(*ε*_r,2,4_), *X*_5_(*ε*_r,2,5_)]; *T*_3_[*X*_1_(*ε*_r,3,1_), *X*_2_(*ε*_r,3,2_), *X*_3_(*ε*_r,3,3_), *X*_4_(*ε*_r,3,4_), *X*_5_(*ε*_r,3,5_)]; *T*_4_[*X*_1_(*ε*_r,4,1_), *X*_2_(*ε*_r,4,2_), *X*_3_(*ε*_r,4,3_), *X*_4_(*ε*_r,4,4_), *X*_5_(*ε*_r,4,5_)]}. If we list the temperatures and mole fractions in ascending order, then the variable space covers a temperature range from *T*_1_ to *T*_4_, and a mole fraction range from *X*_1_ to *X*_5_. If *X* represents the mole fraction for the solvent having the higher relative permittivity, then the variable space covers a relative permittivity range from *ε*_r,4,1_ to *ε*_r,1,5_.

### Derivative expressions and the linearized plots

Analyzing a data set using eqn (1) entails generating a plot for which two system variables (the domain variables) are varied, and the other two are constant. The activation free energy plot can be in terms of either domain variable. The complete set of possible activation free energy plots for a binary solvent includes: 
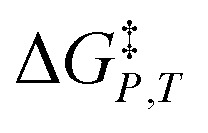
*vs. X* or *ε*_r_, 
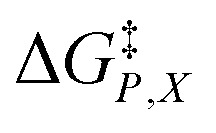
*vs. T* or *ε*_r_, 
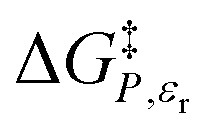
*vs. T* or *X*, 
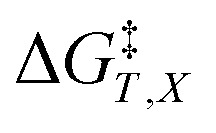
*vs. P* or *ε*_r_, 
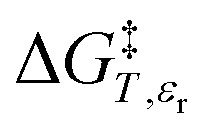
*vs. P* or *X*, and 
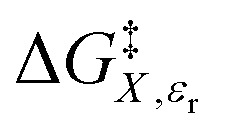
*vs. T* or *P*. For conducting an analysis, a series of plots are generated in a systematic fashion as described in the previous subsection, and the linearization technique, described in the following paragraphs, is applied to each plot in the series.

We will use the isobaric/isothermal expression to illustrate the linearization technique. The domain variables under these conditions include the mole fraction and relative permittivity, and so we will need solvent model equations. To model the electrostatic term, we use the following Kirkwood–Onsager equation:^5,6^9
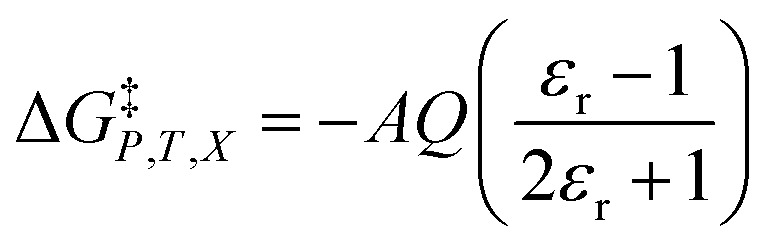
in which *A* is a constant given by:10
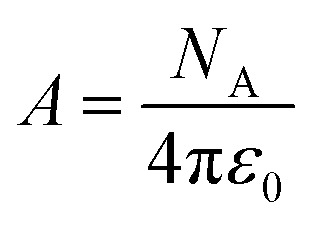
and *Q* is a convenient substitution parameter given by:11
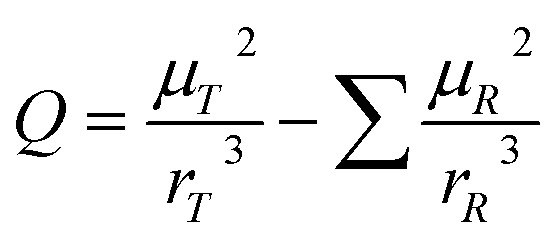
*N*_A_ is Avogadro's number, *ε*_0_ is the vacuum permittivity, *μ*_R_ is the dipole moment for the reactant molecule designated *R*, *r*_R_ is its radius, *μ*_T_ is the transition-state dipole moment, *r*_T_ is its radius, and the summation in eqn (11) is over the number of reactant molecules. An empirical mole fraction equation that can be linear or non-linear, and one that we have used in previous work is:^2–4^12
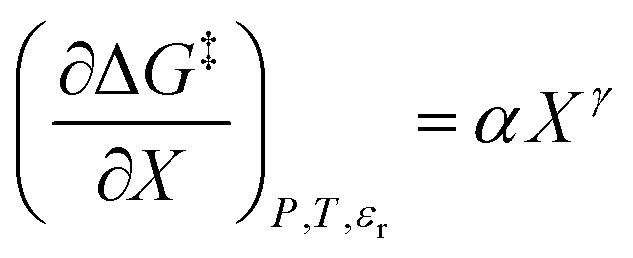
in which *α* and *γ* are the model parameters.

Treating *Q* as a constant, the derivative expression for 
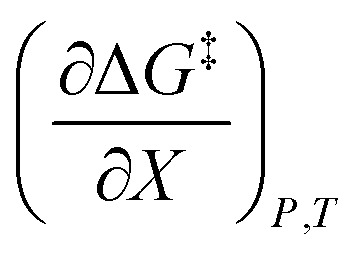
 is:13
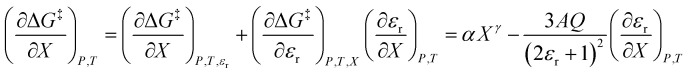


Treating all the solvent model parameters as constants, the integrated equation within limits is:14
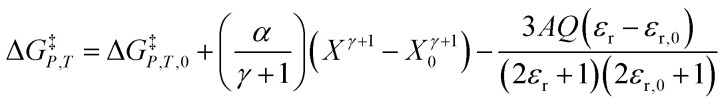
in which the subscript “0” denotes initial values. The mole fraction and relative permittivity are related by the appropriate form of the solvent grid equation, so eqn (14) can be cast solely in terms of either domain variable. Dividing eqn (13) by *X*^*γ*^ yields the following linearized form of the equation:15
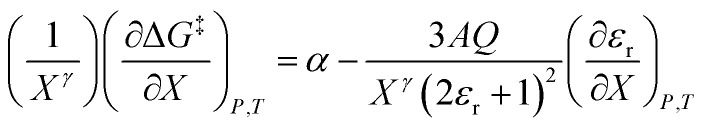


Eqn (15) is analyzed by plotting 
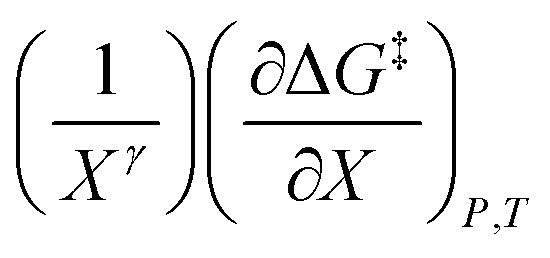
*vs.*
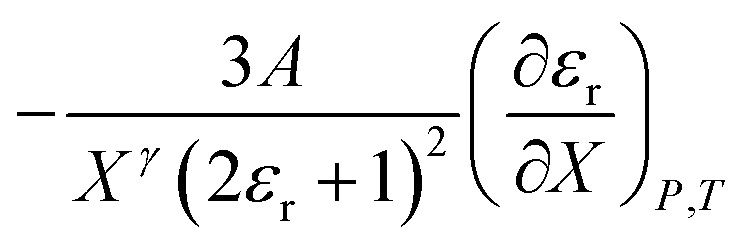
 and conducting a linear fit, for which the slope is *Q* and the intercept is *α*. Expressions for 
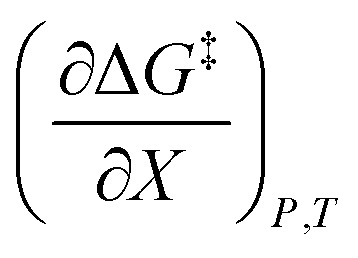
 are determined by fitting the plots of 
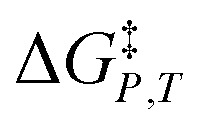
*vs. X* with suitable polynomials and evaluating the derivatives. A suitable polynomial is one that has the lowest order possible and yields a reasonably good fit within the standard deviations of the data points. We avoid higher order polynomials (generally fourth order or higher) as they lead to erratic or oscillatory behavior. The solvent term, 
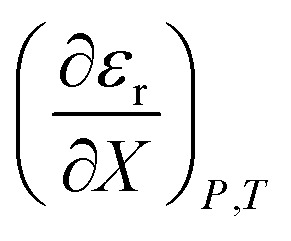
, is evaluated using the appropriate form of the solvent grid equation given by *ε*_r_ = f^*ε*^_*P*,*T*__r_(*X*). A pre-determined value for *γ* is required, and we will discuss how to obtain this in a moment.

In addition to eqn (15), there are five more independent linearized equations. Using eqn (9) and (12) as the solvent models, they are:16
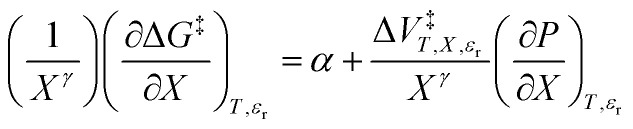
17
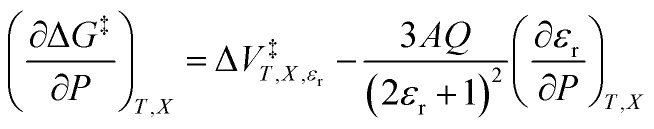
18
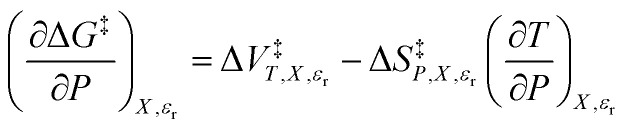
19
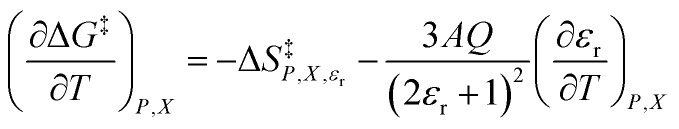
20
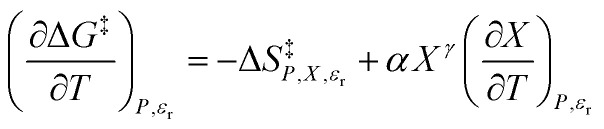


Eqn (16)–(20) are analyzed in the same fashion as eqn (15). The slopes and intercepts from the linear fits of these equations are: *Q* and *α* from eqn (15), 
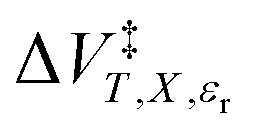
 and *α* from eqn (16), *Q* and 
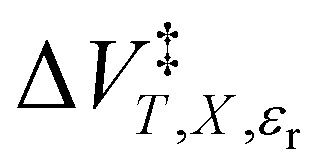
 from eqn (17), 
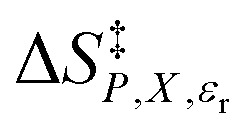
 and 
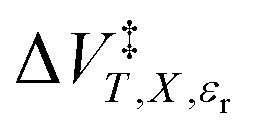
 from eqn (18), *Q* and 
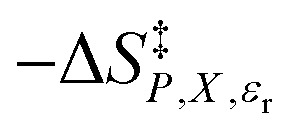
 from eqn (19), and *α* and 
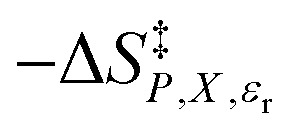
 from eqn (20). If eqn (12) is used to model the mole fraction term, a value for *γ* must be determined before analyzing eqn (15) and (16), or (20). One method for doing this is to conduct a regression analysis using the integrated expression, and either treat *γ* as a fitting parameter or preassign a value that yields good regression results.

### The “two-point” technique for evaluating the functional dependencies in terms of the domain variables

If the plot of the linearized equation is linear, then the parameters are constant. If the plot is not linear, a “two-point” technique, described as follows, can be used to evaluate the functional dependencies for the parameters. Assume the activation thermodynamic and/or solvent model parameters are constant between all sets of adjacent data points. Now consider the representative linear expression, *y* = *p*_i_ + *p*_s_*x*, in which *y* and *x* are the dependent and independent variables, respectively, and *p*_s_ and *p*_i_ are the slope and intercept, respectively. The values for *p*_s_ and *p*_i_ between any two adjacent points (designated 1 and 2) are calculated from the following equations:21
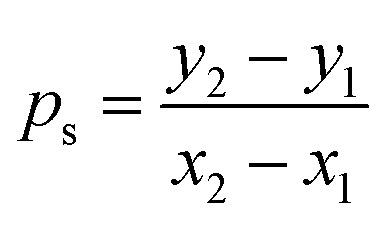

22 *p*_i_ = *y*_1_ − *p*_s_*x*_1_ = *y*_2_ − *p*_s_*x*_2_

The slopes and intercepts from this analysis are those for the midpoint (*i.e. x* = ½(*x*_1_ + *x*_2_) and *y* = ½(*y*_1_ + *y*_2_)), and therefore the (*p*_s_, *p*_i_) data set contains one less pair of terms than the (*x*, *y*) data set used in the analysis. Of course, the parameters are not exactly constant between adjacent points, so tighter experimental grids should improve the precision of the analysis.

To evaluate an integral equation, all parameters that vary or that cannot be factored out must be incorporated into the integrands. Moreover, all variable parameters must be cast in terms of their primary variables. Hence, the proper integral expression for eqn (15) (assuming *γ* is constant) is:23
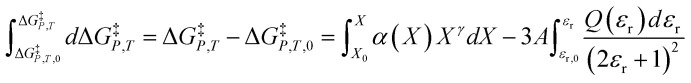


The functional forms for *α*(*X*) and *Q*(*ε*_r_) are determined from polynomial fits of the set of values from the two-point analyses. Generally, the integral expressions are numerically evaluated.

If a parameter is not constant, then it must explicitly depend on either or both domain variables. We can understand this better by investigating all the possible functionalities for one of the parameters. For instance, consider *Q*(*ε*_r_) in eqn (23), for which the differential equation with respect to *ε*_r_ is:24
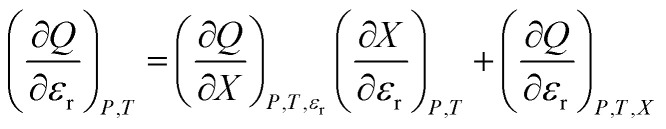


Even though *Q*(*ε*_r_) is cast solely in terms of *ε*_r_, it can still depend on *X.* If *Q* depends solely on *ε*_r_, then only the second right-hand-side term in eqn (24) is non-zero. If *Q* depends solely on *X*, then only the first right-hand-side term, which is implicit in *ε*_r_, is non-zero. If *Q* depends on both variables, as may likely be the case, then both terms are non-zero. We note here that a two-point analysis cannot be used to analyze the intrinsic terms 
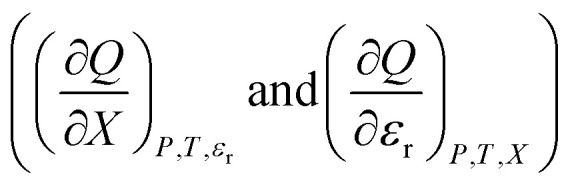
.

The accuracy of a two-point analysis can (and should) be tested by expressing the experimental values for Δ*G*^‡^ and the integrated equation in terms of the same domain variable, and plotting them on the same graph for comparison.

### Using layered polynomials to generate the parameter grid equations

The last step is generating the parameter grid equations, and presenting an example is the best way to describe this step. Suppose we conduct several iso-mole fraction experiments at several temperatures under isobaric conditions and determine that *Q* is not constant, and that second-order polynomials give good fits for the *Q vs. X* plots. The polynomial for the first temperature is:

25 *Q*_1_ = *a*_1_*X*^2^ + *b*_1_*X* + *c*_1_

in which *a*_1_, *b*_1_, and *c*_1_ are the regression parameters. The general expression for any temperature is:

26 *Q*_i_ = *a*_*i*_*X*^2^ + *b*_*i*_*X* + *c*_*i*_

in which *i* goes from one to the number of temperatures. Now suppose the set of fitting parameters represented by *a*_*i*_, *b*_*i*_, and *c*_*i*_ can all be fitted in terms of temperature using second-order polynomials, *i.e.*:27
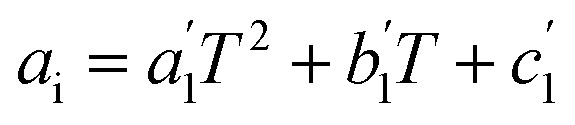
28
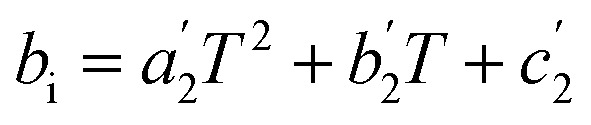
29
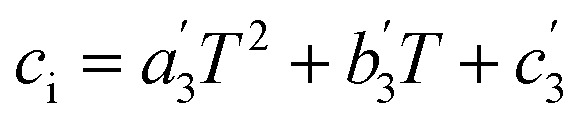


Combining eqn (26) with eqn (27)–(29), and dropping the subscript *i* yields the following isobaric grid equation:30
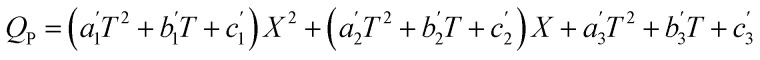


Eqn (30) has two layers of polynomials (the first in *X* and the second in *T*), and applies only within the variable space used in the experimental grid. The parameter grid equations can be put in terms of any two of the system variables using the appropriate forms of the solvent grid equation. For example, *X* can be replaced with *ε*_r_ using the isobaric form of the solvent grid equation given by *X* = f^*X*^_*P*_(*T*,*ε*_r_), for which the expression becomes:31
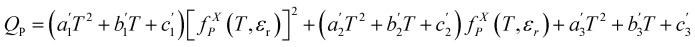
As previously discussed, the polynomial orders should be as low as possible and still yield good regression results. If some other type of equation is used, it should have as few parameters as possible and be as functionally simple as possible. A grid equation is only as precise as the least precise polynomial fit used to generate it, and the polynomial fits are only as precise as the data used in the fits.

### Three-step analysis for the *tert*-butyl chloride hydrolysis reaction in the acetonitrile/water system

**Fig. 1:**
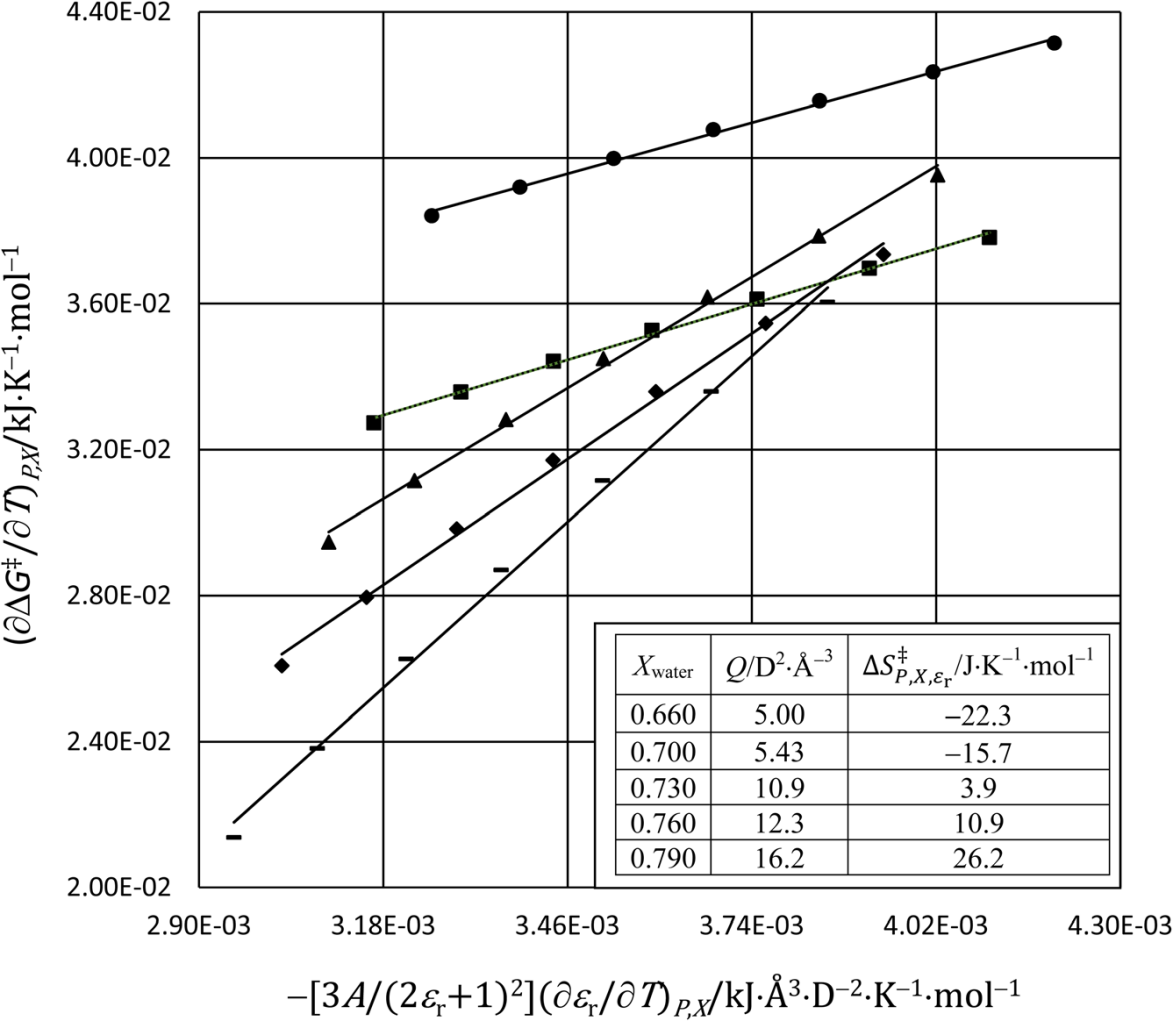
The plots of 
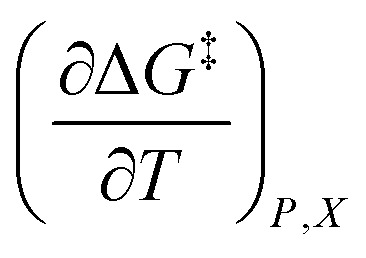
*vs.*
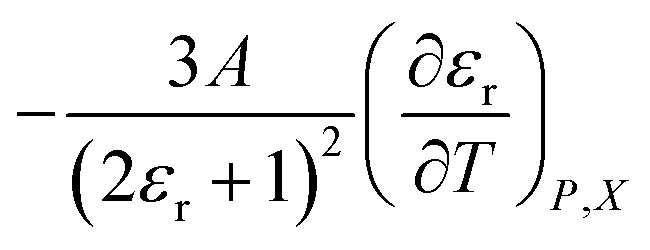
 and linear fits for the hydrolysis of *tert*-butyl chloride in the acetonitrile/water system for the following water mole fractions: 0.660 (●), 0.700 (∎), 0.730 (▲), 0.760 (◆), and 0.790 (−). The rate and solvent data used to calculate the *x*- and *y*-axes terms are in ref. 4. The values for *Q* and 
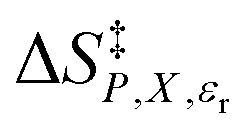
, which are the slopes and intercepts, respectively, are shown in the inset, and the correlation coefficients are all 0.997. D is the Debye unit and Å is an Angstrom. The average values for *Q* and 
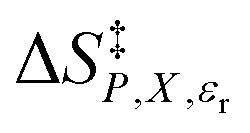
 are 10 D^2^ Å^−3^ and 0.6 J K^−1^ mol^−1^, respectively, in close agreement with the values in ref. 4.

**Fig. 2:**
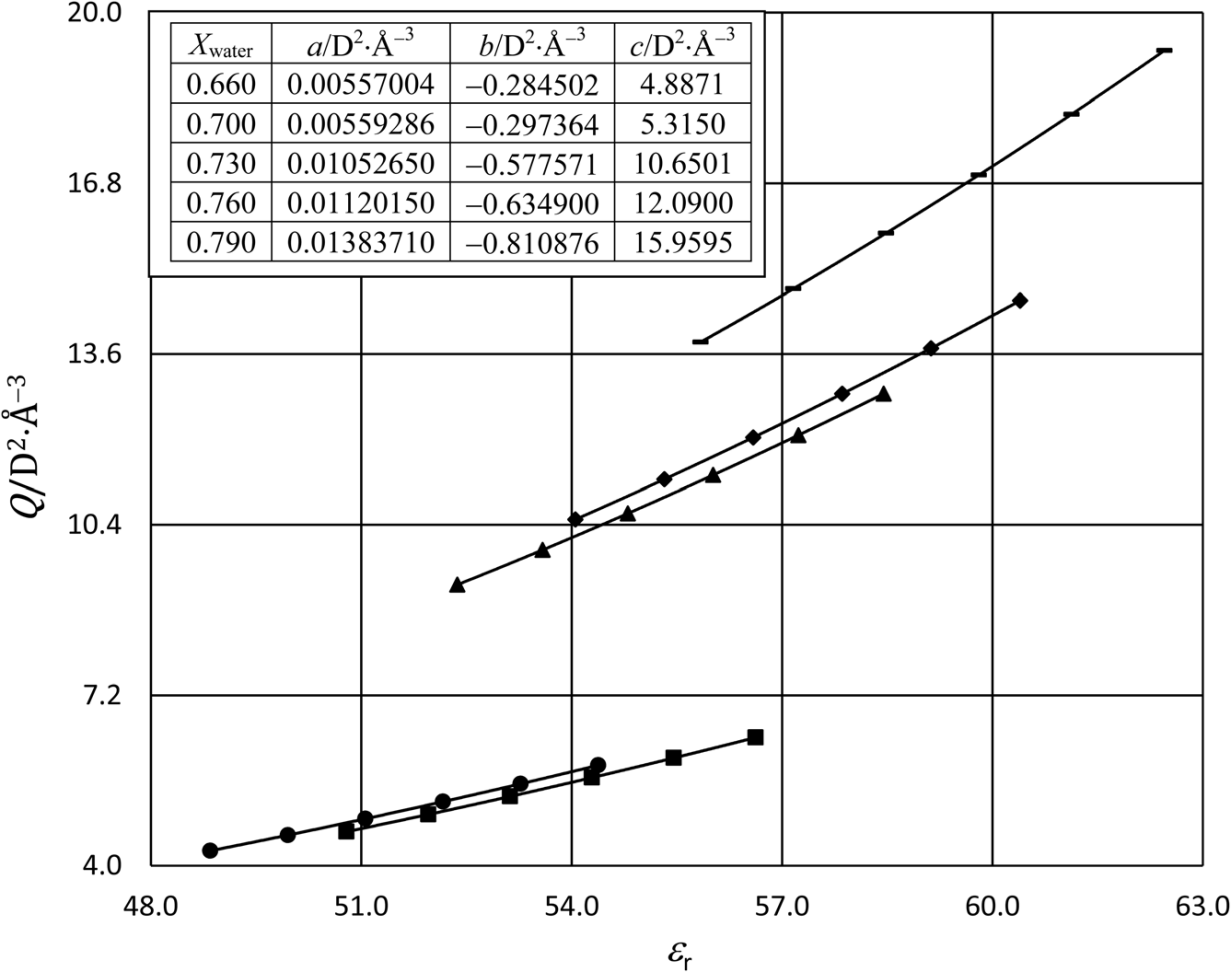
Plots of *Q vs. ε*_r_ using data from the two-point analyses for the following water mole fractions: 0.660 (●), 0.700 (∎), 0.730 (▲), 0.760 (◆), and 0.790 (−). The data is fitted with second-order polynomials (*Q* = *aε*_r_^2^ + *bε*_r_ + *c*). The regression values from the fits are shown in the inset, and the correlation coefficients are all 0.9999999. For each plot, the temperatures range from 22.5 °C for the point furthest to the right to 47.5 °C for the point furthest to the left.

**Fig. 3:**
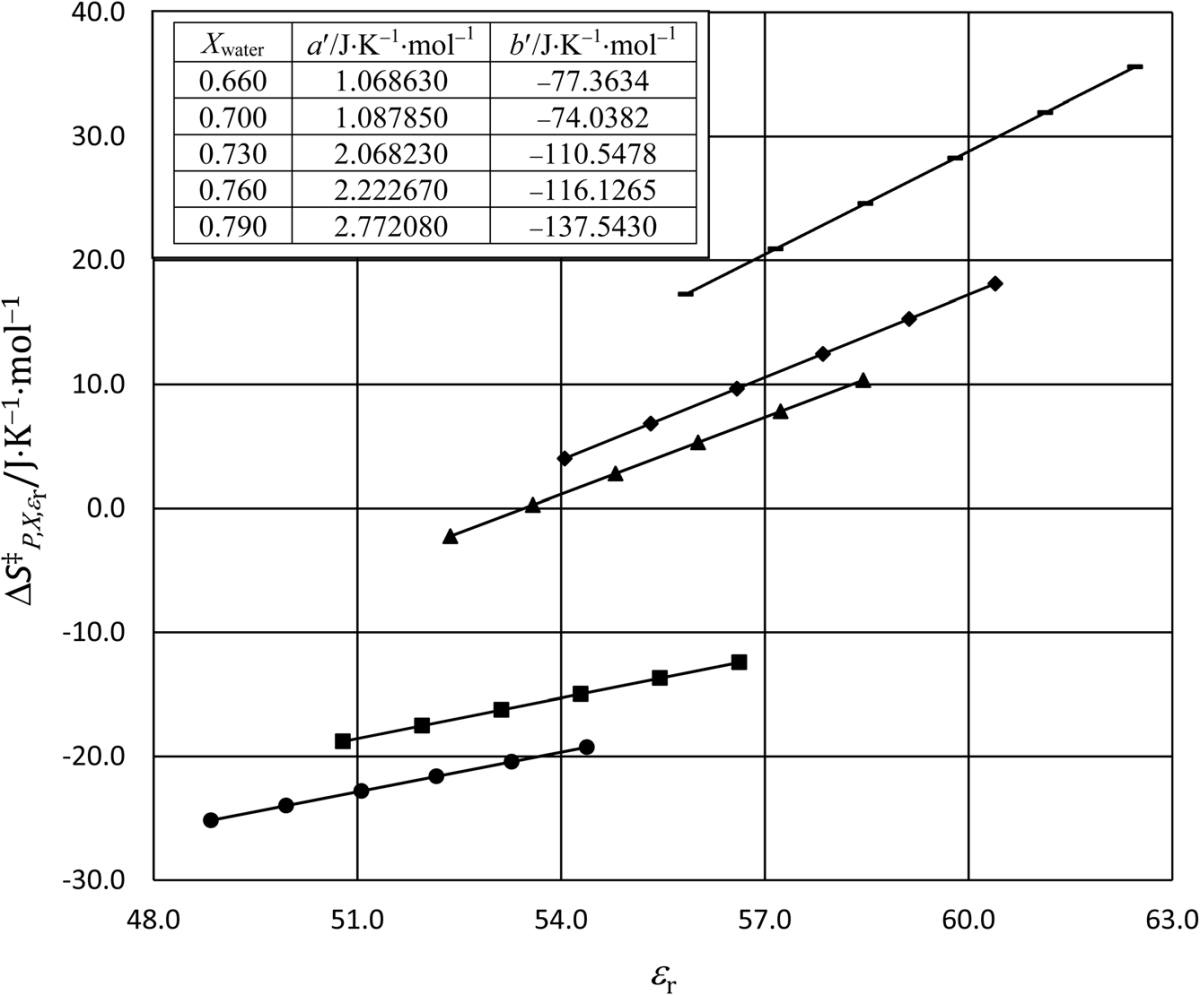
Plots of 
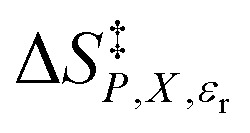
*vs. ε*_r_ using data from the two-point analyses for the following water mole fractions: 0.660 (●), 0.700 (∎), 0.730 (▲), 0.760 (◆), and 0.790 (−). The fits are linear (
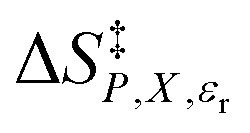
 = *a*′*ε*_r_ + *b*′). The regression values from the fits are shown in the inset, and the correlation coefficients are all 1.00000000. For each plot, the temperatures range from 22.5 °C for the point furthest to the right to 47.5 °C for the point furthest to the left.

In this subsection we show results of the three-step analysis applied to the isobaric/iso-mole fraction rate data from our recent publication.^4^Fig. 1 shows the plots of 
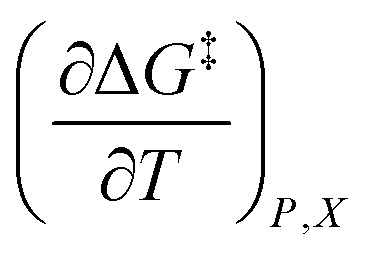
*vs.*
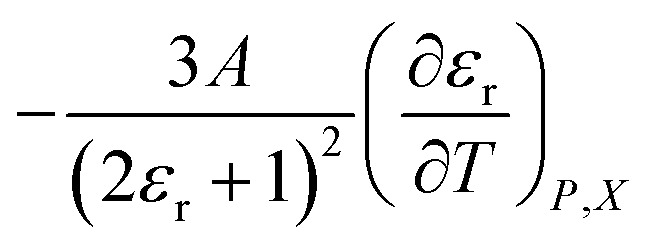
 for five water mole fractions ranging from 0.660 to 0.790. Clearly, the plots are not linear, particularly above *X*_water_ = 0.700. A two-point analysis was conducted for all the data sets and the results are plotted as *Q vs. ε*_r_ and 
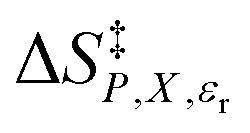
*vs. ε*_r_ in Fig. 2 and 3, respectively. Relative permittivity is chosen for these plots rather than temperature because the parameters are probably more strongly dependent upon relative permittivity, and the functional behaviors of the parameters are more easily explained in terms of relative permittivity. The plots are fitted using suitable polynomials, and the regression values are shown in the insets in Fig. 2 and 3. The integral equation incorporating these polynomials, in which *ε*_r_ is replaced with *ε*_r_(*T*) in the temperature integral, is:32
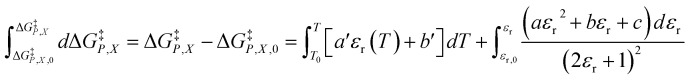


**Fig. 4:**
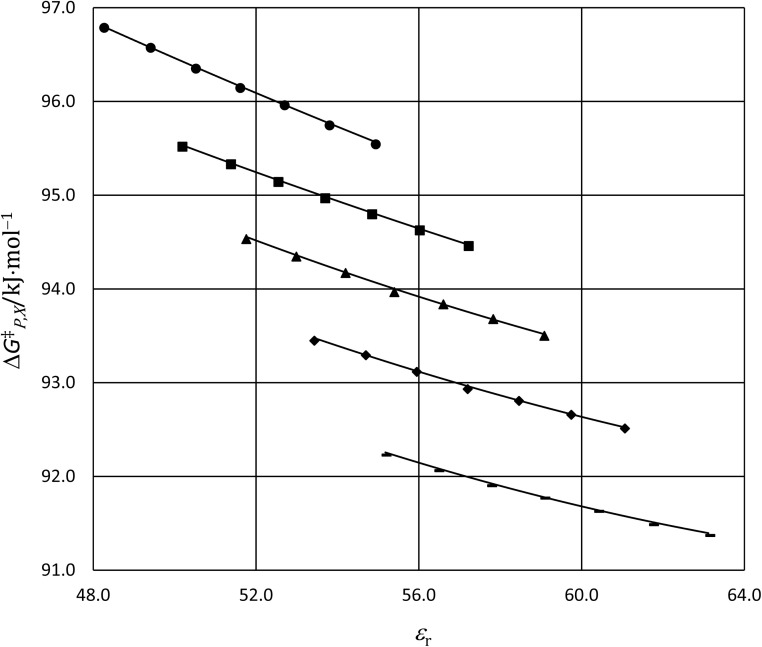
Plots of 
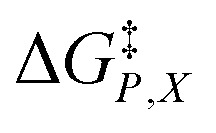
*vs. ε*_r_ and graphs of the integrated equations from eqn (32) for the hydrolysis of *tert*-butyl chloride in the acetonitrile/water system for the following water mole fractions: 0.660 (●), 0.700 (∎), 0.730 (▲), 0.760 (◆), and 0.790 (−). For each plot, the temperatures range from 20.0 °C for the points furthest to the right to 50.0 °C for the points furthest to the left.

The functional forms for *ε*_r_(*T*) are given in ref. 4. Fig. 4 shows the graphs of the integrated equations, which are analyzed numerically, along with plots for the activation free energies from ref. 4. As can be seen, the graphs track the data plots quite well.

Abrupt changes in the slopes and vertical (*y* axis) positions of the plots are observed at certain mole faction intervals in Fig. 1–3. These abrupt changes are reflected in the polynomial regression constants shown in the insets in Fig. 2 and 3. As these constants cannot be fitted well with polynomials (in *X*_water_) lower than fourth order, and fourth order polynomials lead to oscillatory behavior, accurate parameter grid equations cannot be generated for this system. But on the upside, these sudden shifts have some intriguing ramifications that we discuss below.

As s side note, the activation thermodynamic and solvent model parameters have not been constant for the reaction systems we have studied so far. Not enough reactions and solvent systems have been analyzed to determine if this trend is general, but if it is, the implications may be game changing. Activation parameters are traditionally treated as constant in routine analyses. However, if these parameters are not generally constant for most reaction systems, as we strongly suspect may be the case, then a paradigm shift in how condensed-phase rate data is analyzed and interpreted may be in order.

Although we will not delve deeply into the implications of the results presented here, as this is not the primary intent of this article, we do make a few observations to illustrate the insight possible with a three-step analysis. As we have already identified, the plots in Fig. 1–3 exhibit sudden shifts within certain mole fraction intervals. These shifts do not necessarily suggest there are discontinuous changes occurring in the solvent structure at certain mole fractions. It is more likely that significant, but monotonic structural changes are occurring within very narrow intervals at certain mole fractions. The fact that the sudden shifts in the slopes and the vertical positions occur concurrently for *Q* and 
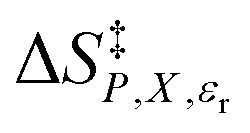
 shows these parameters are subject to the same solvent effect. Spectroscopic evidence indicates there are significant structural changes that occur at certain mole fractions for the acetonitrile/water system,^7–10^ but supposedly very little change occurs within the mole fraction range used in our studies. This suggests that the mole fraction interval effects on *Q* and 
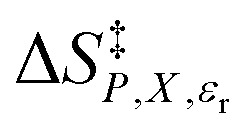
 are not associated with structural changes occurring in the bulk solvent.

The following discussion briefly explores possible explanations for the positive slopes of the plots, and the sudden shifts that occur in the slopes and vertical positions at certain mole fraction intervals. Variability in the activation thermodynamic and solvent model parameters indicates structural changes are occurring in the transition state as the solvent changes. We can illustrate this by considering the behavior of *Q* in Fig. 2. *Q* increases with the transition-state dipole moment (eqn (11)), and therefore correlates with the degree of the charge separation and C–Cl bond length in the transition state. Hence, the monotonic increase of *Q* with relative permittivity as depicted in Fig. 2 reflects these transition-state structural changes.

The effects from mole fraction changes are not nearly as smooth. Specifically, small shifts in *Q* occur between *X*_water_ = 0.660 and 0.700, and between 0.730 and 0.760, but much larger shifts occur between 0.700 and 0.730, and between 0.760 and 0.790. The larger shifts are not due to electrostatic effects, as is evident from the plots in Fig. 2, but instead to very sensitive structural changes within the solvation shell. The transition state may become significantly more solvated with water molecules, or the solvation-shell molecules may reorient more efficiently within these intervals.^11–13^ Either of these can stabilize the developing charges and cause the C–Cl bond to lengthen. As seen by comparing Fig. 2 and 3, the large shifts in *Q* within these mole fraction intervals correlate with the large shifts in 
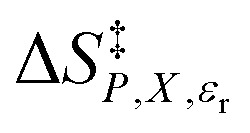
. These changes are accompanied by corresponding increases in 
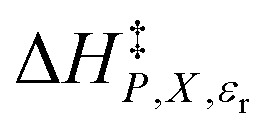
, so that 
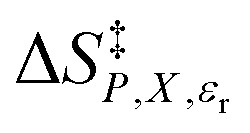
 and 
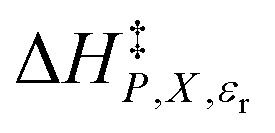
 largely compensate each other. This is reflected in the nominal effects of the mole fraction on 
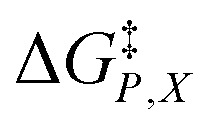
 as indicated in Fig. 4. As *X*_water_ increases, the C–Cl bond lengthens and the transition state becomes more structurally similar to the intermediate state (the separated carbocation and chloride ion). The fact that the shifts in *Q* and 
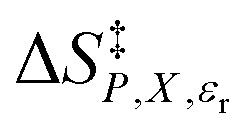
 between 0.760 and 0.790 are smaller than those between 0.700 and 0.730 is consistent with this idea.

Correlations among the activation entropy, enthalpy, and free energy are well documented for many types of reactions,^11–16^ but for equations such as eqn (1) that include solvent model terms, correlations can include solvent model parameters as well. The tight correlation between *Q* and 
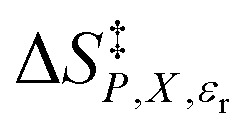
 serves to illustrate this. Among other things, these close correlations undergird the interpretations that we draw from the analysis, and create confidence in the efficacy of the analysis.

### Comparison with the traditional analysis

**Fig. 5:**
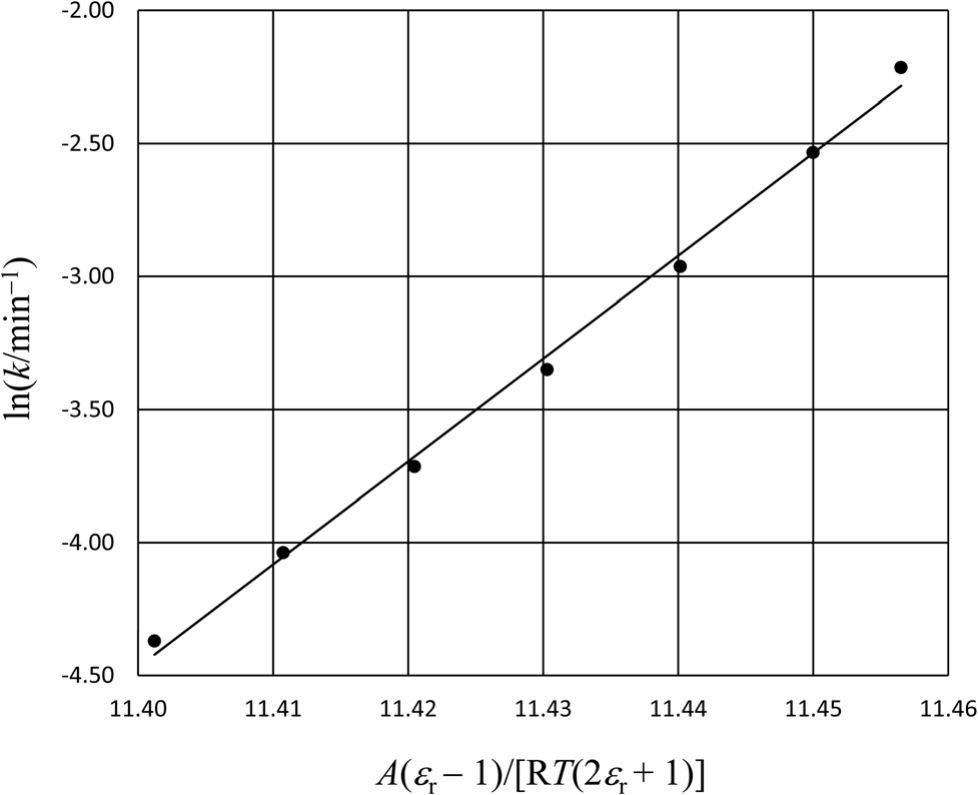
The plot of ln(*k*) *vs.*
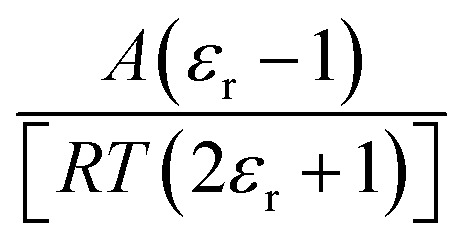
 and the linear regression analysis using eqn (33) for the hydrolysis reaction of *tert*-butyl chloride in the acetonitrile/water solvent system under isobaric/isothermal conditions. The temperature is 35.0 °C, and the water mole fraction/relative permittivity ranges from 0.620/49.7 to 0.790/59.1. The slope from the regression analysis is 38.6 D^2^ Å^−3^, the intercept is −445, and the correlation coefficient is 0.996.

Equating the activation free energy to the electrostatic free energy leads to the following traditional expression^11,17^33
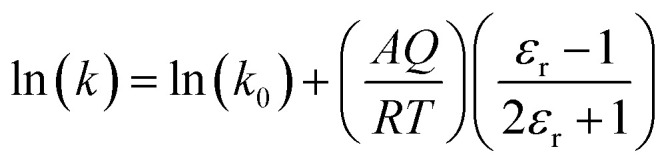
in which *k*_0_ is the rate constant for *ε*_r_ = 1. Rate data is analyzed by plotting ln(*k*) *vs.*
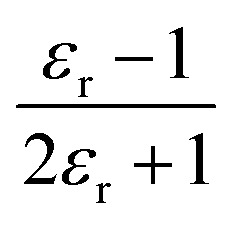
 (the Kirkwood function). Fig. 5 shows the plot for the isobaric/isothermal data from ref. 4 and the linear regression analysis using eqn (33). Clearly, the plot is not linear, which in fact is generally the case for these types of plots.^18,19^ Moreover, the slope (*Q*) for the plot is 38.6 D^2^ Å^−3^, which is unrealistically large.

It is common knowledge that eqn (33) does not account for the effect of the solvation shell. Hence, we expect isobaric/isothermal plots of ln(*k*) *vs.*
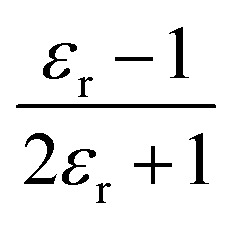
 to be linear only in the case that the solvation shell has little or no effect on the reaction. However, any reaction that is affected by the bulk electrostatic environment is almost assuredly affected by the solvation shell as well, and so an analysis using eqn (33) is not generally expected to lead to meaningful results.

Most plots of ln(*k*) *vs.*
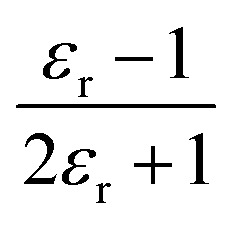
 for reactions in single and binary solvent systems exhibit extensive scatter and very poor correlations of the slope with the Kirkwood function.^20–24^ Moreover, the conventional thought is the transition-state dipole moment is constant for a given reaction in a wide range of solvents. We have shown that for the hydrolysis of *tert*-butyl chloride, *Q* is quite sensitive to the solvent environment. We expect this to be a general trend at least for reactions in which the ionic characters of the reactant and transition states are different.

Given all that we have presented in this article, we submit that rate data in binary solvents cannot be accurately analyzed apart from eqn (1).

## Summary

The activation parameters associated with the fundamental equation of chemical kinetics may or may not be constant over an experimental variable space. Due to compensatory effects of the system variables on the reaction rates, obtaining good regression results when the parameters are kept constant is not an indication that these parameters are actually constant. This article introduces a novel three-step technique that systematically assesses whether the parameters are constant, and provides a method for evaluating the functional dependencies if they are not. This technique includes: (1) determining if the data follows a linear trend using the regression results from a linearized form of the fundamental equation, and if not, (2) evaluating the activation parameters between all sets of adjacent points in the data set, and if possible, (3) constructing parameter grid equations over the experimental variable space.

The fundamental equation contains intrinsic solvent terms that uniquely account for the bulk-phase electrostatic effect, and for the close–range interactions associated with the solvation shell. These terms are used to model solvent equations. Unlike empirical equations, the parameters associated with theoretic equations, such as the Kirkwood–Onsager equation, have structural significance. Evaluating the functional dependencies for these parameters, now made possible by the three-step technique introduced in this article, can provide much deeper insight into the solvent-dependent structural features of the reaction system.

As a proof of concept, we applied the three-step technique to some of the rate data for the hydrolysis of *ter*t-butyl chloride in the acetonitrile/water binary system presented in a recent publication. The analysis showed strong functional dependencies for the Kirkwood–Onsager parameter and the intrinsic activation entropy, and provided clear evidence for the correlation between the solvent relative permittivity and the C–Cl bond length in the transition state. The analysis also revealed some interesting effects from the solvation shell that could not have been uncovered without the three-step analysis.

## Data availability

The data used for the analyses presented in this article are available upon request. Refer all inquiries to Dr. Floyd L. Wiseman, at fwiseman@bmc.edu.

## Conflicts of interest

There are no conflicts of interest to declare.

## Supplementary Material

RA-015-D4RA07211A-s001
