# Supplementary material for: A technique for analyzing the variability of activation thermodynamic and solvent model parameters
Source: RSC Adv. 2025 Feb 6;15(6):4111–9. doi: 10.1039/d4ra07211a (PMC11801181; doi:10.1039/d4ra07211a)
Supplement: RA-015-D4RA07211A-s001 [file RA-015-D4RA07211A-s001.pdf]

Supplementary Information (SI) for RSC Advances.
This journal is © The Royal Society of Chemistry 2025

A technique for analyzing the variability of activation thermodynamic and solvent model parameters

Floyd L. Wiseman[a*], Dane W. Scott[b]
[a]Blue Mountain Christian University Department of Mathematics & Natural Science; P.O. Box 160, Blue Mountain, MS 38610
[b]East Tennessee State University Department of Chemistry; 325 Treasure Lane; Johnson City, TN 37614

Keywords: Fundamental thermodynamic equation of chemical kinetics, activation free energy, binary solvents, activation thermodynamic and solvent model parameters, intrinsic terms, parameter functional dependencies, differential solvation

Supplemental Sections

1 A Closer Look at the Intrinsic Terms in the Fundamental Equation..S3

2 Comparisons with the Conventional Equation..S4

3 Deriving a Relationship that Correlates the Mole Fraction Term with the Solute-Solvent Interactions Associated with the Solvation Shell..S5

1 A Closer Look at the Intrinsic Terms in the Fundamental Equation

We explore the possibility of obtaining mathematical expressions for the intrinsic terms. The motivation is that these terms cannot be independently measured since no single system variable can be varied while the other three remain constant. In our quest, we will consider the following differential isobaric/isothermal expression from Equation (1):

$$\left(\frac{\partial \Delta G^{\ddagger}}{\partial \varepsilon_r}\right)_{P,T} = \left(\frac{\partial \Delta G^{\ddagger}}{\partial \varepsilon_r}\right)_{P,T,X} + \left(\frac{\partial \Delta G^{\ddagger}}{\partial X}\right)_{P,T,\varepsilon_r}\left(\frac{\partial X}{\partial \varepsilon_r}\right)_{P,T} \tag{1.1}$$

$\left(\frac{\partial \Delta G^{\ddagger}}{\partial \varepsilon_r}\right)_{P,T}$ and $\left(\frac{\partial X}{\partial \varepsilon_r}\right)_{P,T}$ both can be evaluated from experimental data, leaving the intrinsic solvent model terms, $\left(\frac{\partial \Delta G^{\ddagger}}{\partial \varepsilon_r}\right)_{P,T,X}$ and $\left(\frac{\partial \Delta G^{\ddagger}}{\partial X}\right)_{P,T,\varepsilon_r}$, as unknowns in the equation. To obtain a sufficient number of equations for our analysis, we add the following independent equations:

$$\left(\frac{\partial \Delta G^{\ddagger}}{\partial T}\right)_{P,X} =- \Delta S^{\ddagger}_{P,X,\varepsilon_r} + \left(\frac{\partial \Delta G^{\ddagger}}{\partial \varepsilon_r}\right)_{P,T,X}\left(\frac{\partial \varepsilon_r}{\partial T}\right)_{P,X} \tag{1.2}$$

$$\left(\frac{\partial \Delta G^{\ddagger}}{\partial X}\right)_{P,\varepsilon_r} = \left(\frac{\partial \Delta G^{\ddagger}}{\partial X}\right)_{P,T,\varepsilon_r} - \Delta S^{\ddagger}_{P,X,\varepsilon_r}\left(\frac{\partial T}{\partial X}\right)_{P,\varepsilon_r} \tag{1.3}$$

We now have three equations and three unknowns, namely $\left(\frac{\partial \Delta G^{\ddagger}}{\partial \varepsilon_r}\right)_{P,T,X}$, $\left(\frac{\partial \Delta G^{\ddagger}}{\partial X}\right)_{P,T,\varepsilon_r}$, and $- \Delta S^{\ddagger}_{P,X,\varepsilon_r}$ (which is $\left(\frac{\partial \Delta G^{\ddagger}}{\partial T}\right)_{P,X,\varepsilon_r}$). We can solve the equations for any one of these. Solving for $\left(\frac{\partial \Delta G^{\ddagger}}{\partial \varepsilon_r}\right)_{P,T,X}$ yields the following rather unwieldy expression:

$$\left(\frac{\partial \Delta G^{\ddagger}}{\partial \varepsilon_r}\right)_{P,T,X}\left(\frac{\partial \varepsilon_r}{\partial T}\right)_{P,X} - \left(\frac{\partial \Delta G^{\ddagger}}{\partial T}\right)_{P,X} = \left(\frac{\partial \varepsilon_r}{\partial T}\right)_{P,X}\left(\frac{\partial X}{\partial \varepsilon_r}\right)_{P,T}\left(\frac{\partial \Delta G^{\ddagger}}{\partial X}\right)_{P,\varepsilon_r} - \left(\frac{\partial \varepsilon_r}{\partial T}\right)_{P,X}\left(\frac{\partial \Delta G^{\ddagger}}{\partial \varepsilon_r}\right)_{P,T} + \left(\frac{\partial \varepsilon_r}{\partial T}\right)_{P,X} \tag{1.4}$$

We immediately see that the terms containing $\left(\frac{\partial \Delta G^{\ddagger}}{\partial \varepsilon_r}\right)_{P,T,X}$ cancel, dashing our hopes of finding an expression for $\left(\frac{\partial \Delta G^{\ddagger}}{\partial \varepsilon_r}\right)_{P,T,X}$.

Solving for the other two intrinsic terms or performing a similar analysis using another set of independent equations all render the same results. But while this analysis is rather disappointing, it is pedagogically useful as we have proven that mathematical expressions for intrinsic terms cannot be obtained.

2 Comparisons with the Conventional Equation

The conventional equation for the activation free energy at constant pressure and mole fraction is[1]:

$$\Delta G_{P,X}^{\ddagger} = \Delta G_{P,X,0}^{\ddagger} - \Delta S_{P,X,0}^{\ddagger}(T - T_0) - T\Delta C_{P,X}^{\ddagger}\left(\ln\frac{T}{T_0} + \frac{T_0}{T} - 1\right) \tag{2.1}$$

We can compare this equation with the following fundamental isobaric/iso-mole fraction equation, in which the intrinsic activation heat capacity term has been added:

$$\Delta G_{P,X}^{\ddagger} = \Delta G_{P,X,0}^{\ddagger} - \Delta S_{P,X,\varepsilon_r,0}^{\ddagger}(T - T_0) - T\Delta C_{P,X,\varepsilon_r}^{\ddagger}\left(\ln\frac{T}{T_0} + \frac{T_0}{T} - 1\right) - \frac{3AQ(\varepsilon_r - \varepsilon_{r,0})}{(2\varepsilon_r + 1)(2\varepsilon_{r,0} + 1)} \tag{2.2}$$

In the following paragraphs we discuss the fundamental differences associated with $\Delta C^{\ddagger}_{P,X,\varepsilon_r}$ and $\Delta C^{\ddagger}_{P,X}$, and the benefits of Equation (2.2).

We begin with a rather bold statement, namely that the intrinsic activation heat capacity term is unlikely to make significant contributions for most reactions. In one of our early articles, we presented an equation (not shown here) that relates the activation heat capacity to the suite of reactant and transition state modes[2]. From this equation we can determine that for unimolecular reactions there is essentially no contribution from the translational and internal rotational modes. For bimolecular reactions the contribution from these modes is about 25 $J \cdot K^{-1} \cdot mol^{-1}$, which accounts for ~0.1 $kJ \cdot mol^{-1}$ change in activation free energy over a 50°C range. The contributions from the internal and intermolecular vibrational modes are difficult to estimate, but probably contribute no more than ~0.13 $kJ \cdot mol^{-1}$ over a 50°C range. We base this estimate on the difference in molar heat capacities between liquid water and ice. The conclusion here is these numbers are negligible unless the change in the activation free energy over a 50°C range is ~4 $kJ \cdot mol^{-1}$ or less.

Reported values for $\Delta C^{\ddagger}_{P,X}$ are almost always larger than our estimates for $\Delta C^{\ddagger}_{P,X,\varepsilon_r}$, which is not surprising since $\Delta C^{\ddagger}_{P,X}$ accounts for changes in relative permittivity as well as temperature. However, literature values for $\Delta C^{\ddagger}_{P,X}$, notably those for enzymatic reactions, range from ~1 to ~20 $kJ \cdot K^{-1} \cdot mol^{-1}$.[2-6] We believe these numbers are unreasonably large. To be fair, we have used Equation (1.1) in some of our work, but either for comparative purposes[7], or before we introduced the fundamental equation to the scientific community.[8, 9] Several researchers have in fact raised concerns and objections over these inordinately large activation heat capacities.[10-12]

The intrinsic activation heat capacity and electrostatic terms in Equation (1.2) both can accommodate nonlinear behavior, making this equation quite versatile for modeling curvature. $\Delta G^{\ddagger}_{P,X}$ vs. T plots generally exhibit minimal curvature for non-enzymatic reactions, so the use of both nonlinear terms may be hard to justify. In this case the electrostatic term is probably more defensible. Even for enzymatic reactions, for which the plots are substantially more curved, electrostatic effects are likely responsible for much of the curvature. If it is deemed that the intrinsic activation heat capacity may be significant for an enzymatic system, then both terms can be used in the analysis.

3 Deriving a Relationship that Correlates the Mole Fraction Term with the Solute-Solvent Interactions Associated with the Solvation Shell

The following analysis correlates the mole fraction term in Equation (1) with the close-range solute-solvent interactions associated with the solvation shell. Differential solvation,

defined as the difference in the mole fraction between the solvation shell and the bulk solvent[13], forms the bedrock for this analysis.

Figures 3.1 and 3.2 show representative graphs for the solvent mole fraction vs. the radial distance from the reactant molecule and transition state, respectively. These graphs illustrate the variation of the mole fractions within the solvation shell, which may be several molecular diameters deep. At some radial distance, represented by r^{RSbulk} and r^{TSbulk}, the mole fraction becomes that of the bulk solvent, which is the same for both graphs. The activation free energy associated with the influence of the solvation shell is given as:

$$\Delta G^{\ddagger}_{P,T,\varepsilon_r} = G^{TS}_{P,T,\varepsilon_r}\left(X^{TS}_0\right) - G^{RS}_{P,T,\varepsilon_r}\left(X^{RS}_0\right) \quad (3.1)$$

in which the right-hand-side terms are those for the mole fraction at the inner layer of the

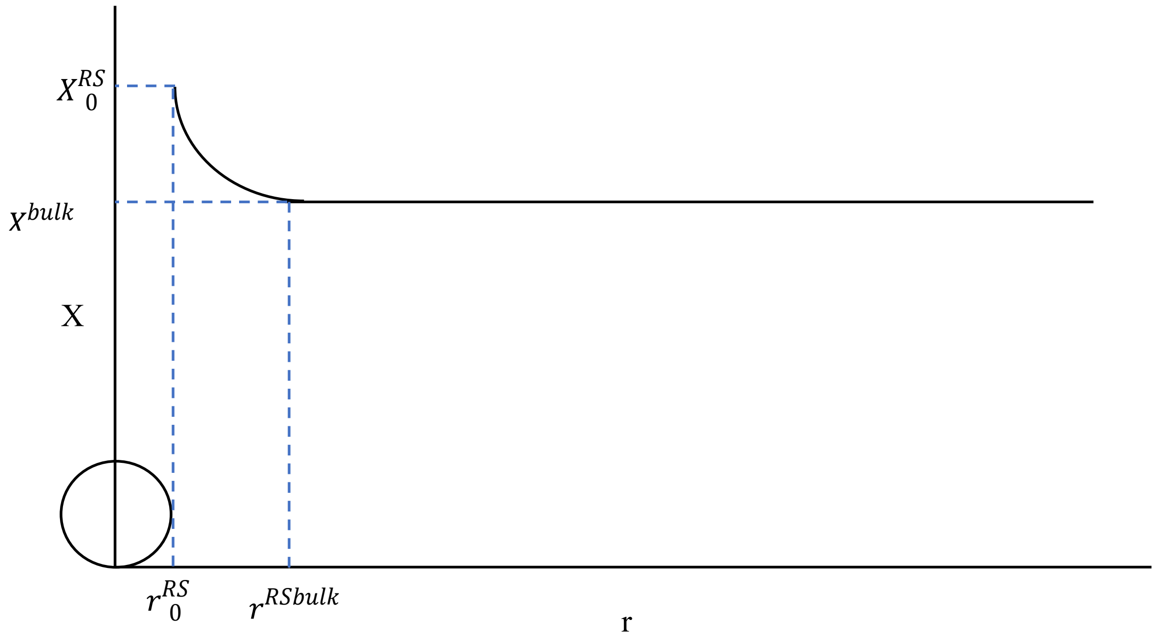

Figure 3.1. Graph of the solvent mole fraction vs. the radial distance (r) from the center of the reactant molecule having a radius r^{RS}_0 (RS refers to the reactant state). X^{RS}_0 is the mole fraction for the inner layer of the solvation shell, and X^{bulk} is the bulk-phase mole fraction. The depth of the solvation shell is $r^{RSbulk} - r^{RS}_0$, and r^{RSbulk} is the radial distance at which the mole fraction becomes X^{bulk}.

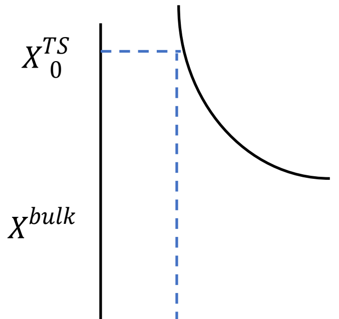

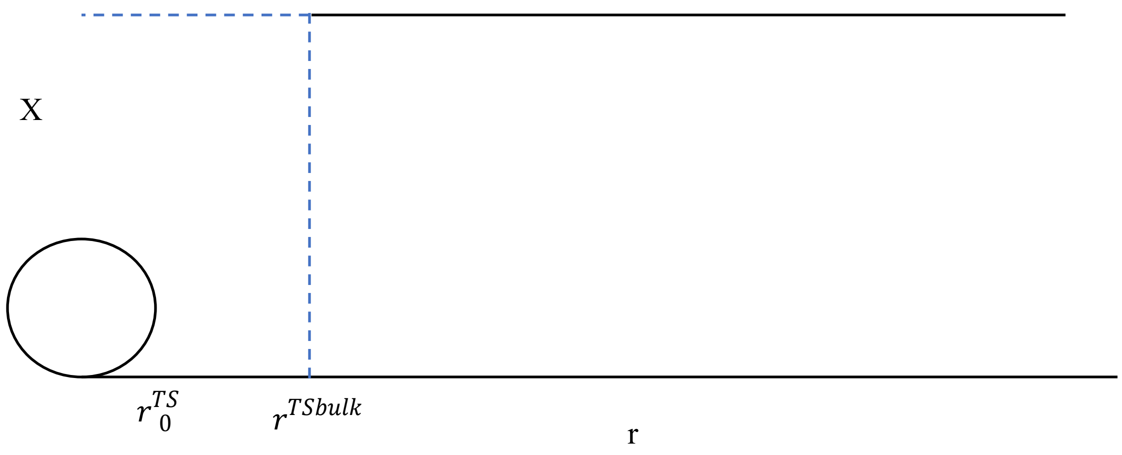

Figure 3.2. Graph of the solvent mole fraction vs. the radial distance from the center of the transition state having a radius r_0^{TS}. X_0^{TS} is the mole fraction for the inner layer of the solvation shell, and X^{bulk} is the bulk-phase mole fraction. The depth of the solvation shell is $r^{TSbulk} - r_0^{TS}$.

solvation shells. Note that in general, $r^{TS}_{0} \neq r^{RS}_{0}$, $r^{TSbulk} \neq r^{RSbulk}$, and $X^{TS}_{0} \neq X^{RS}_{0}$. The mole fraction term evaluated from Equation (3.1) is:

$$\left(\frac{\partial \Delta G^{\ddagger}}{\partial X}\right)_{P,T,\varepsilon_r} = \left(\frac{\partial G^{TS}}{\partial X}\right)_{P,T,\varepsilon_r}\left(X^{TS}_{0}\right) - \left(\frac{\partial G^{RS}}{\partial X}\right)_{P,T,\varepsilon_r}\left(X^{RS}_{0}\right) \tag{3.2}$$

in which *X* represents the bulk-phase mole fraction, and the first and second right-hand-side terms are evaluated at X^{TS}_{0} and X^{RS}_{0}, respectively. Equation (3.2) can be written implicitly as:

$$\left(\frac{\partial \Delta G^{\ddagger}}{\partial X}\right)_{P,T,\varepsilon_r} = \left(\frac{\partial G^{TS}}{\partial X^{TS}_{0}}\right)_{P,T,\varepsilon_r}\left(\frac{\partial X^{TS}_{0}}{\partial X}\right)_{P,T,\varepsilon_r} - \left(\frac{\partial G^{RS}}{\partial X^{RS}_{0}}\right)_{P,T,\varepsilon_r}\left(\frac{\partial X^{RS}_{0}}{\partial X}\right)_{P,T,\varepsilon_r} \tag{3.3}$$

$\left(\frac{\partial G^{TS}}{\partial X^{TS}_{0}}\right)_{P,T,\varepsilon_r}$ and $\left(\frac{\partial G^{RS}}{\partial X^{RS}_{0}}\right)_{P,T,\varepsilon_r}$ represent free energy changes at the inner layer of the solvation shells, and $\left(\frac{\partial X^{TS}_{0}}{\partial X}\right)_{P,T,\varepsilon_r}$ and $\left(\frac{\partial X^{RS}_{0}}{\partial X}\right)_{P,T,\varepsilon_r}$, which are differential solvation terms, represent the inner layer mole fraction variations with the bulk-phase mole fraction. These differential solvation terms directly correlate the mole fraction term, $\left(\frac{\partial \Delta G^{\ddagger}}{\partial X}\right)_{P,T,\varepsilon_r}$, with the effect of the solvation-shell inner layers.

Supplemental References

1. E. C. W. Clarke and D. N. Glew, *Transactions of the Faraday Society*, 1966, **62**, 539-547.
2. E. J. Walker, C. J. Hamill, R. Crean, M. S. Connolly, A. K. Warrender, K. L. Kraakman, E. J. Prentice, A. Steyn-Ross, M. Steyn-Ross, C. R. Pudney, M. W. van der Kamp, L. A. Schipper, A. J. Mulholland and V. L. Arcus, *ACS Catalysis*, 2024, **14**, 4379-4394.
3. V. L. Arcus and A. J. Mulholland, *Annual Review of Biophysics*, 2020, **49**, 163-180.
4. R. S. Firestone, S. A. Cameron, J. M. Karp, V. L. Arcus and V. L. Schramm, *ACS Chemical Biology*, 2017, **12**, 464-473.
5. V. L. Arcus, E. J. Prentice, J. K. Hobbs, A. J. Mulholland, M. W. Van der Kamp, C. R. Pudney, E. J. Parker and L. A. Schipper, *Biochemistry*, 2016, **55**, 1681-1688.

6. M. Oliveberg, Y. J. Tan and A. R. Fersht, *Proc Natl Acad Sci U S A*, 1995, **92**, 8926-8929.
7. F. L. Wiseman and D. W. Scott, *Physical Chemistry Chemical Physics*, 2024, DOI: 10.1039/D3CP04155G.
8. W. C. C. Floyd L. Wiseman, Curtis Heishman, Brandon Robinson, *International Journal of Advanced Research in Chemical Science*, 2015, **2**, 1-9.
9. F. L. Wiseman, *Journal of Physical Organic Chemistry*, 2012, **25**, 1105-1111.
10. J. Åqvist and F. van der Ent, *Journal of Chemical Theory and Computation*, 2022, **18**, 6345-6353.
11. J. Åqvist, J. Sočan and M. Purg, *Biochemistry*, 2020, **59**, 3844-3855.
12. S. Roy, P. Schopf and A. Warshel, *J Phys Chem B*, 2017, **121**, 6520-6526.
13. A. Ben-Naim, *Pure and Applied Chemistry - PURE APPL CHEM*, 1990, **62**, 25-34.
